# Vigor at work mediates the effect of transformational and authentic leadership on engagement

**DOI:** 10.1038/s41598-022-20742-2

**Published:** 2022-10-12

**Authors:** Esther Lopez-Zafra, Manuel Pulido-Martos, Daniel Cortés-Denia

**Affiliations:** Departamento de Psicología. Área de Psicología Social, Edif. C5, Universidad de Jaén, Campus Las Lagunillas s/n, 23071 Jaén, Spain

**Keywords:** Psychology, Human behaviour

## Abstract

Several studies have posited that authentic leadership (AL) and transformational leadership (TFL) imply ethical behaviour that can mitigate tendencies towards low engagement at work. However, there is a lack of studies analysing, for the same sample, the effect of both styles as a job resource and their effects on employees’ engagement as a means of facilitating their work goals and reducing their job demands. This study addresses this shortcoming by analysing the relations of both leadership styles to vigor, an affective construct, and engagement at work, a motivational outcome. Moreover, the possible mediation effect of vigor at work on the relationship between both leadership styles and engagement is considered. Finally, we explore the differential contributions of both styles to employees’ resources. A sample of Spanish employees (*N* = 215; 48.8% female) under the supervision of a direct leader responded concerning the TFL and AL of their closest supervisor and their own vigor at work and engagement. Our results show that vigor increases the effect of both leadership styles on engagement. Moreover, TFL, to a greater extent than AL, relates to higher engagement. Thus, vigor as an affective dimension mediates the effect of positive leadership on engagement. This study considers, for the same sample, the effects of two related, albeit different, leadership styles. The results indicate that AL and TFL are positively perceived by employees as creating a climate of energy that acts as a resource (both organizational and personal). Practitioners could enhance employees’ vigor at work and engagement by promoting these two leadership styles.

## Introduction

Only 20% of employees were engaged at work in 2020^1^. Leaders need to address this problem to reduce its negative consequences (i.e., low retention, low job performance). A focus on positive aspects by organizations could help to invert this tendency. One positive aspect is leadership. Leaders have the responsibility to attain the group goals and thus, they strive to obtain, maintain, protect and promote different resources (material/economic, social or energetic) that would be highly valued by their employees. The conservation of resources (COR) theory^2,3^ indicates that resources can be transmitted through positive experiences and states, and this implies crossover effect (direct or indirect). In our study, we focus on leadership as leaders maybe the major transmitters of energy and positivity. In fact, leadership is considered to be a ”positive aspect in the organizational context that influences employees’ thriving^4^^(p. 149)”^. Thus, recent research has focused on proposing emerging leadership styles that are considered to be positive leadership styles^5^, including moral or ethical leadership (ethical, authentic, and servant leadership^6^) or modern leadership styles that have a clear impact on positive results (transformational leadership^7^). Moreover, a positive leadership style approach has been proven to relate positively to different outcomes, such as enhancing employee well-being, increasing individual performance and organizational productivity, or increasing organizational citizenship behaviour (see the systematic review by Malinga et al.^7^). However, there still exists much confusion regarding the construct of positive leadership in the literature, since it is a combination of several other positive forms of leadership (ethical, servant, authentic, and transformational^7^). In all cases, the ethical issue of leadership is behind the positive aspect of leadership, and, thus, some results could be compared, but studies tend to focus on one of the approaches.

The job demands-resources theory (JD-R), expands the COR theory by considering the joint participation of job *demands* (i.e. physical, psychological, social or organizational aspects that imply an effort^8^) and *resources* (i.e. physical, psychological, social, or organizational aspects that help to achieve a goal, to reduce demands, or to stimulate personal development^9,10^) in obtaining health and motivational outcomes (i.e. engagement).

Leadership could be considered a positive resource that helps to reduce the job demands, or as a construct that influences other demands and resources including personal resources. Thus, leadership is recently considered a construct at a superior level (see meta-analysis on this debate^11^) that has an impact on personal resources and outcomes as engagement, that is, a construct influencing health or performance.

**Figure 1:**
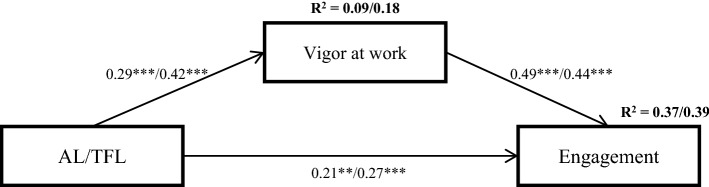
Mediation models. **p < 0.01; ***p < 0.001. The AL coefficient values are on the left side of the bar, and those of TFL are on the right side. AL = Authentic leadership; TFL = Transformational leadership.

In this study, we consider the impact that two positive leadership styles may have on the sense of energy that employees might feel. This energy is conceptualized by vigor at work, which is an affective state that could act as a personal resource, and engagement, which is well-grounded as a motivational outcome in the JD-R theory (see Fig. 1. in the results section).

The two leadership styles that have received the most attention in research are the most positively oriented: authentic leadership (AL) and transformational leadership (TFL)^12^. This study focuses on comparing these two positive leadership styles, to better capture their differing contribution to employees ‘sense of energy.

TFL was proposed in the 1980s, and since then, it has been the most widely studied and advocated form of leadership^13^. Bass^14,15^ explained that TFL represents moral and ethical leadership that serves the good of the group, organization, or country and does not harm followers^16^. In essence, it has been suggested that leaders motivate followers to work beyond their own expectations and help them achieve high performance, inspiring high levels of involvement in the group^17^. TFL has proved to be the most effective form of organizational leadership, and meta-analytic reviews have reported on average moderately strong relationships between TFL and subordinate performance^18,19^, suggesting that increases in TFL might result in improvements in employee performance.

Its effect occurs through motivational processes of the transformational leader that generates a collective identity, trust in the team, vision of the future, among others. In addition, it provides support and confidence that generate a reduction of stress preventing negative mental states^20^. Therefore, in the JD-R theory, the TFL is mainly analysed as a factor that influences the variables that produce health effects^21^. In summary, the results to date show that the TFL influences both resources and work demands, producing changes in both positive and negative indicators of employee well-being^22^. In short, the TFL generates labour resources in the form of autonomy, social support, and feedback that increase engagement levels and, at the same time, allows to face the demands of work (for a revision^23^).

However, recent studies have suggested that TFL might have peaked in developed economies (i.e., Western Europe and North America), while it is still most effective in developing countries (i.e., Sub-Saharan Africa, the Middle East, Southern Asia, and Latin America)^24^. Among the reasons for this decrease in the interest on TFL is that subordinates in developed countries may be particularly suspicious of leaders because of prior negative experiences with pseudo-transformational leaders, who only seek to fulfil their own self-interest^16,25,26^. This implies questioning TFL^27^.

In contrast, authentic transformational leadership “must rest on a moral foundation of legitimate values”^28^^(p. 184)^, and the violation of this ethical requirement by a leader is named pseudo-transformational leadership^29^.

In the need for ethical and an authentical leadership, this specific proposal resulted in a new positive leadership style: AL. It is defined as the “process that draws from both positive psychological capacities and a highly developed organizational context, which results in both greater self-awareness and self-regulated positive behaviours on the part of leaders and associates, fostering self-development”^30^^(p. 243)^. It has been argued that AL produces a personal and social feeling of identification in followers and establishes an elevated moral standard and high values^31^. Moreover, these leaders are interested in empowering their followers to make a difference^32^. AL theory has received empirical support from the management literature^33^. The interest in AL has quickly flourished in management literature^34^, but, at the same time, this leadership style is also put into question due to its conceptual ambiguity and measurement^35,36^.

This debate about the groundings of different positive leadership styles makes it even more necessary to clarify which is the differential conceptualization and contribution of each other. At the conceptual level, both styles focus on the development of followers as a way to reach and exceed goals, although they differ in mechanisms of influence. Authentic leaders are not based on charisma, inspiration, or the use of symbols to build strong and lasting relationships with their followers, but on their behaviour, dedication, and exemplary and transparent conduct^37^. In sum, TFL and AL are similar in terms of self-confidence, hopefulness, resilience, and high moral standards. However, TFL strives to make organizational changes, whereas authentic leaders only promote changes as role models^38^. Empirically, TFL and AL are competing styles in recent research, and several studies have compared them with different results^31^. In fact, meta-analyses indicate that there may be some construct redundancy^38,39^. However, most studies have focused on the antecedents of these leadership styles or their effects on performance, and further analyses concerning their impact on employees´ resources are needed.

In this sense, employees’ perceptions of these leadership styles would be related to higher perceptions of employees’ energy (Hypothesis 1), facilitating their work goals and reducing their job demands. Thus, we consider that AL and TFL leaders can transmit energy in a crossover effect that could enhance employees´ resources. The question now is: how can we conceptualize this energy?

### Consequences of perceived leadership style on employees’ “energy”: Vigor at work and engagement

In organizational psychology, several constructs refer to "employee's energy", such as vitality, thriving, enthusiasm, vigor at work and engagement. Regarding the last two, empirical research has shown they are two distinct constructs^40^. Additionally, a systematic review performed by Cortés-Denia et al.^41^ showed their differential contributions to health and thus, they could also differ in other aspects.

Considering the COR and the JD-R approaches^42^ work engagement is a motivational process resulting from the interaction between demands and resources; whereas vigor at work can be regarded as a personal resource that derives from the work experiences related to feelings of energy that are individually possessed^43^. In this venue, vigor at work could be considered as a personal resource that could have an effect on different results^44^. Thus, vigor at work could be an antecedent of engagement which is an outcome of several resources (i.e. job resources as leadership and personal resources as vigor at work).

Vigor at work derives from the COR theory and refers to individuals' feelings that they possess physical strength, emotional energy, and cognitive liveliness, and represents a moderate-intensity affect experienced at work^43^. This positive affective state is generated by continuous interactions among different elements in the workplace and refers to a higher extent to social and physical personal resources. As mentioned, is characterized by physical strength, which refers to the individual’s physical abilities; emotional energy, which is the ability to express sympathy, empathy, and compassion to others; and cognitive liveliness, which pertains to mental agility and the flow of thought processes^43,45^. Hence, vigor at work reflects workers' feelings concerning the energy reserves they have at work, and is closely related to motivational processes in the workplace^46^. In this vein, motivation is the result of the collection of energy forces that determine the direction, intensity, and duration of initiating and maintaining work-related behaviour^47,48^. In this sense, vigor at work can be considered a prerequisite or precursor of work motivation^46^ and, therefore, of work engagement.

Work engagement is a positive and emotional-motivational state in workers and it has been considered an indicator of well-being at work^49^. Its importance lies in the results it obtains such as its effect on work performance, as well as its positive effects on the well-being of workers (i. e., less negative symptoms or increased job satisfaction)^50^. It is composed of three dimensions. The first is the vigor dimension which is different from vigor at work previously explained as an engagement dimension. This vigor dimension of engagement is characterized by energy levels, but it also includes mental resilience, effort, and persistence at work. The other two dimensions are dedication, which refers to inspiration, enthusiasm, challenge, and pride; and absorption, which represents a state of concentration and immersion in a task^49^. This construct has been widely studied and has produced consistent results related to reducing negative results and enhancing positive results^41^. Moreover, leadership is identified as a promotor of work engagement^51^.

Thus, vigor at work may be a potential mediator and could intervene in the relationship between the AL/TFL and work engagement, given the implication of leadership on employees’ energy, and on motivational variables. In this vein, vigor at work could be a fundamental underlying process or mechanism in this relationship (Hypothesis 2).

Moreover, we consider that the contribution of both leadership styles could be different. Specifically, TFL includes how a leader inspires followers, changes cognitions, and pushes followers to a higher level of motivation, empowering them to see and achieve goals^52^. In this sense, vigor at work and engagement are considered to be highly related to TFL.

Studies show that this relation applies to engagement^53^, which acts as a mediator of TFL on performance^54^. Moreover, engagement, in particular, has been viewed as an important consequence of AL^55^, in which employees who perceive their supervisors to be authentic leaders are more engaged in their work^56^, as proposed by the theoretical framework of Avolio and Gardner^57^. Finally, the positive impact of AL on work engagement depends on how subordinates perceive their leaders^58,59^. In sum, several studies concerning TFL and AL, separately, have shown these styles to predict and/or increase employee engagement^60–64^.

However, studies that consider high levels of energy in terms of vigor usually use the vigor dimension at the engagement scale. For example, Tafvelin et al.^65^ focused on the leader’s vigor, as measured by the Utrecht Work Engagement Scale (UWES^66^), and suggested that high levels of energy are a beneficial resource for leaders, as such energy enables them to promote transformation and reduce employee burnout. However, the analysis of vigor at work as a personal resource, different from the vigor subdimension of the engagement construct, is still a venue to be analysed in depth. To our knowledge, only Wouters et al.^67^ analysed, and found, a positive relationship between vigor and AL using Shirom’s concept of vigor to address this issue.

In summary, it has been well established that leadership determines employee work engagement^68,69^. However, no studies are comparing the two styles of leadership in terms of providing a job resource (TFL and AL), a personal resource (vigor at work), and a motivational result (work engagement). Thus, we explore the differential contributions of TFL and AL to these employees´ resources. Specifically, we claim that TFL instils higher engagement and vigor at work than does AL (Hypothesis 3).

## Method

### Participants and procedure

A sample of 215 employees working in different organizations from Spain (48.8% female) voluntarily participated in this study (*M* = 39.4 years; *SD* = 11.99; range 19–65 years). Participants were employees in their organization for at least three months and worked under the supervision of a direct leader. The average tenure in the organization was 9.39 years (*SD* = 10.15; range 0.25–46 years). The organization sectors were 67% private, 30.7% public, and 2.3% mixed.

Employees from different organizations were contacted for participation, and those who consented were approached in their workplace by psychology undergraduate students involved in a research seminar. The students were instructed on the procedure and distribution of the survey, following the protocol approved by the Ethics Committee of the University of Jaén, Spain (Ref. NOV.19/1.PROY). This procedure follows the guidelines provided for applying this sampling technique^70,71^. The design of the study was quantitative and correlational research. Participants responded to a questionnaire that included the variables of interest described in the measures section. Informed consent was obtained from all participants.

### Measures

#### Transformational leadership

Participants completed a short version of the Multifactor Leadership Questionnaire (MLQ)^72^ in Spanish^73^, in which employees evaluated their perceptions of the leadership style of their supervisors. Of a total of 22 items, we used the 13 items that measured TFL, with a five-point Likert response format ranging from 1 (highly unlikely) to 5 (highly likely). These items addressed the four components of TFL: (a) Charisma or Idealized Influence (4 items), (b) Inspirational Motivation (3 items), (c) Intellectual Stimulation (3 items), and finally (d) Individualized Consideration (3 items). However, we used the global TFL score due to very strong correlations among dimensions (all correlations were between 0.85 and 0.93, indicating strong and very strong positive relations^74^) to better capture the overall leadership style.

#### Authentic leadership

This variable was assessed using the Authentic Leadership Questionnaire (ALQ^75^; Spanish version by Moriano et al.^76^). The Spanish version comprises 13 items with a five-point Likert response format ranging from 0 (highly unlikely) to 4 (highly likely). This version contains four subscales: (a) Relational Transparency (3 items); (b) Internalized Moral Perspective (3 items); (c) Balanced Processing of Information (3 items); and (d) Self-Awareness (4 items). The Spanish version found that these four first-order factors were also grouped as second-order factor. This finding was in agreement with Walumbwa et al.´s^75^ results, and in this study, we use the one-factor solution to compare with TFL.

#### Vigor at work

The Shirom-Melamed Vigor Measure (SMVM^45^; Spanish version by Pulido-Martos et al.^77^) was used to measure vigor at work. This scale contains 12 items provided with a 7-point Likert response format (ranging from 1 = almost never to 7 = almost always) and comprises three subscales: Physical Strength (5 items), Cognitive Liveliness (3 items), and Emotional Energy (4 items).

#### Work engagement

The Utrecht Work Engagement Scale (UWES-17 by Schaufeli et al.^49^) was used to measure engagement. This 17-item scale employs a 7-point Likert response format (ranging from 1 = almost never to 7 = almost always) and comprises three dimensions: Vigor (6 items), Dedication (5 items), and Absorption (6 items). In this study, engagement is considered in general, and thus, it is appropriate to evaluate in a unidimensional model^78^.

#### Sociodemographic

The workers informed researchers of their gender, age, sector (public/private/mixed), and tenure in the organization.

### Data analyses

The IBM SPSS statistical package v.26 was used to compute descriptive statistics (means, standard deviations, and Pearson correlations) and exploratory factor analysis. AMOS v.24 was used to analyse the reliability, convergent, and discriminant validity among measures and EQS 6.1 software was used for the confirmatory factorial analysis and the goodness of fit of the models, through the robust method of maximum likelihood. The indicators χ^2^ S-B (Satorra–Bentler chi-square), CFI (comparative fit index), NFI (normed fit index), NNFI (non-normed fit index), and RMSEA (root mean square error of approximation) were used to test the fit of the models. The values greater than 0.90 for CFI, NFI and NNFI indicate an acceptable model fit^79^, meanwhile values less than 0.08 are considered an acceptable model fit in RMSEA^80,81^. PROCESS v. 3.4^82^ with a 95% confidence interval (CI), 5,000 bootstraps, and Davidson–Mackinnon’s heteroscedasticity-consistent inference were used to perform mediation models. Gender, age, sector, and tenure in the organization were controlled in all analyses.

### Ethical approval

All procedures performed in the study involving human participants were under the ethical standards of the institutional and the national research committee (Approved by the Ethics Committee of the University of Jaén, Spain: Ref. NOV.19/1.PROY) and with the 1964 Helsinki declaration and its later amendments or comparable ethical standards.

### Informed consent

All the participants explicitly consented to participate in the study.

## Results

### Descriptive analysis

Table 1 shows the means, standard deviations, and correlations among all variables in the study. The Pearson correlation coefficients showed that AL and TFL were positively related to vigor and engagement at work. Similarly, vigor at work was positively related to work engagement (Hypothesis 1).

**Table 1:** Descriptive statistics, correlations, reliability, convergent and discriminant validity values.

	Mean	SD	CR	AVE	1	2	3	4
1. AL	2.39	1.01	0.941	0.798	(0.894)			
2. TFL	3.37	1.11	0.965	0.874	0.847***	(0.935)		
3. Vigor at work	5.38	0.90	0.767	0.526	0.277***	0.384***	(0.726)	
4. Engagement	4.23	1.10	0.912	0.775	0.339***	0.414***	0.550***	(0.880)

AL = Authentic leadership; TFL = Transformational leadership; SD = Standard deviation; CR = Composite reliability; AVE = Average variance extracted.

The value in parentheses presents the square root of AVE.

***p < 0.001.

### Exploratory and confirmatory factorial analysis, convergent and discriminant validity among measures

First, the main criticism about AL and TFL is that they measure similar aspects of leadership and, further, researches of engagement consider that vigor at work is part of engagement, as it includes a dimension named vigor. Therefore, we conducted exploratory factor analysis that allows us to identify the common factors that explain the order and structure among measured variables^83^. Thus, the results of these analyses could help us to (not)be distorting final assumptions about being measuring the same constructs, and, finally to better capture how items from the two constructs are loaded and grouped in this sample. Therefore, we computed two separate analyses: one for all the items in the two leadership style scales and one for the vigor and engagement scales. The results showed that the items did not overlap in the same construct and that the loadings were congruent with the original instruments. Specifically, for the leadership styles scales (TFL and AL) the principal component extraction method was applied. The Kaiser–Meyer–Olkin (KMO) sample adequacy measure was calculated, indicating a value of 0.96, and Bartlett´s test was statistically significant (χ^2^_120_ = 5991.10; *p* < 0.001), and thus, making the application of factor analysis pertinent. The Varimax rotation procedure was used.

The dimensional structure obtained was composed of two factors that coincided with the two leadership styles. A factor included the items for the TFL instrument proposal (except for item 13) and the other factor included all the AL items and included the item 13 from the TFL instrument. These two factors together explain 70.21% of the variance. The first factor explains 64.60% of the variance corresponding to the AL style and obtains an internal consistency index of 0.96 both in Cronbach´s alpha and the McDonald´s Omega. The second factor corresponds to the TFL style and explains 5.61% of the variance. Item 13 saturated in both leadership styles. The item refers to “my leader likes to introduce changes in the company” and thus, could be perceived by the participants as a global behaviour not dependent on one of these specific styles. Thus, we decided to discard it from subsequent analyses. The overall consistency index of the instrument reached 0.97 in Cronbach´s alpha and McDonald´s Omega.

For the vigor and engagement scales we followed the same procedure. The principal component extraction method was applied. The (KMO) sample adequacy measure was calculated, indicating a value of 0.91, and Bartlett´s test was statistically significant (χ^2^_120_ = 4878.746; *p* < 0.001), and thus, making the application of factor analysis pertinent. The Varimax rotation procedure was used.

The dimensional structure obtained was composed of five factors that explained together the 69.51% of the variance. The first factor explained the 41.99% of the variance and included all the items for the engagement construct except for the items 1, 12, 16 and 17 of the UWES instrument. This factor obtains an internal consistency index of 0.94 both in Cronbach´s alpha and the McDonald´s Omega. The items 12, 16 and 17 loaded in a fifth factor that accounted for the 3.92% of the variance and related to the persistence to continue working. These items reflect the resilience aspect of engagement as proposed by Shirom^45^ and thus we decided to maintain them. However, the UWES item 1 loaded in the second factor that corresponds to the "physical strength" dimension proposed in the vigor at work instrument. This item says “in my work, I feel full of energy” which is the same as the item 1 in the vigor scale which says “I feel full of energy at work”. This second factor accounted for the 11.54% of the variance, with a Cronbach's alpha and a McDonald´s Omega of 0.95. Thus, as the statistical loadings and the construct significance was higher for the vigor at work construct, we decided to discard item 1 from the engagement scale. The third factor explains 6.98% of the variance and saturates the items corresponding to the "emotional energy" dimension of vigor at work SMVM, achieving an internal consistency of 0.87 in Cronbach´s alpha and 0.88 in McDonald´s Omega. Finally, the fourth factor accounted for the 5.07% of the variance and the items loading this factor were related to the cognitive liveness of the SMVM vigor at work construct, achieving an internal consistency of 0.91 in Cronbach´s alpha and 0.92 in McDonald´s Omega. The overall consistency index of the instrument reached 0.92 in Cronbach´s alpha and McDonald´s Omega. Thus, except for item 1, the items for the engagement and the vigor at work constructs did not overlap in the same dimensions, indicating that they are different constructs.

Additionally, the adjustment values of the confirmatory factorial analyses were carried out for four different models (see Table 2). The models included were of a single-factor structure (Model 1); two-factor structure (both leadership styles in one factor and both vigor and engagement in another factor; Model 2); three-factor structure (AL factor, TFL factor, and vigor and engagement in another factor; Model 3); and finally four-factor structure (AL, TFL, vigor at work and work engagement; Model 4). The results yielded the worst rates for Model 1 and 2. The differences in χ^2^ S-B when compared between model 3 and 4 were significant. Model 4 had a better fit to the data (χ^2^ S-B = 138.85; df = 71; *p* < 0.001; CFI = 0.97; NFI = 0.94; NNFI = 0.96; RMSEA = 0.07[0.05–0.08]).

**Table 2:** Adjustment values for the tested models.

	Χ^2^ S-B	df	*p*	CFI	NFI	NNFI	RMSEA	90% CI of RMSEA	Δ χ^2^ S-B
Model 1	689.64	77	< 0.001	0.73	0.71	0.68	0.19	0.18–0.21	–
Model 2	325.08	76	< 0.001	0.89	0.86	0.87	0.12	0.11–0.14	− 4154.12
Model 3	264.02	74	< 0.001	0.92	0.89	0.90	0.11	0.10–0.12	63.88***
Model 4	138.85	71	< 0.001	0.97	0.94	0.96	0.07	0.05–0.08	98.76***

Model 1: A single-factor structure; Model 2: Two-factor structure (leadership styles factor and energy factor); Model 3: Three-factor structure (leadership styles, vigor at work and engagement); Model 4: Four-factor structure (TFL, AL, vigor at work and engagement).

CFI = Comparative fit index; df = Degrees of freedom; CI = Confidence interval; NFI = Normed fit index; NNFI = Non-normed fit index; RMSEA = Root mean square error of approximation; χ^2^ S-B = Satorra–Bentler chi-square; Δ χ^2^ S-B = Scaled Satorra–Bentler chi-square difference.

***p < 0.001.

Finally, convergent validity and reliability were evaluated using the composite reliability (CR), and average variance extracted (AVE)^84^, given correlations are very high among the variables studied. These results were adequate, obtaining results over 0.70 for CR and values over 0.50 for AVE^84,85^. The discriminant validity among factors was assessed through the square root of the AVE^86^, where these values must be higher than the correlations of all the constructs^87^. The results of the square root of the AVE of the variables met this requirement (see Table 1). Considering both exploratory and validity analyses we could assert that there is no overlap among the constructs and thus, they are measuring different aspects.

### Mediation analyses

Simple mediation analyses were carried out to test the effect of AL and TFL independently on work engagement via vigor at work (Hypothesis 2). Thus, Macro PROCESS was used (Model 4^82^), in which AL and TFL were analysed as predictor variables, work engagement as a variable result (outcome), and vigor at work as a possible mediator for both models. By incorporating them separately we avoid the jointed interaction that could emerge due to some construct redundancy^38,39^, and the possible common method bias. Note that employees responded to both leadership styles of their leader and thus, they could have a clear notion of their leader behaviours that are most representative of them.

The results showed a significant direct effect of AL on work engagement (*β* = 0.21, SE = 0.07, *p* = 0.002, CI 95% [0.087, 0.366]) and a significant total effect of AL on work engagement, which was even stronger when considering vigor at work (*β* = 0.35, SE = 0.08, *p* < 0.001, CI 95% [0.226, 0.539]). In the same way, the direct effect of TFL on work engagement was significant (*β* = 0.27, SE = 0.07, *p* < 0.001, CI 95% [0.130, 0.410]), and the total effect via vigor at work was also significant (*β* = 0.46, SE = 0.07, *p* < 0.001, CI 95% [0.311, 0.594]). Regarding the indirect effect, these models show significant relationships via vigor at work (Hypothesis 2) because zero was not included in the 95% CI, but the effect has more weight in the context of TFL (*β* = 0.18, SE = 0.04, 95% CI [0.111, 0.265]) than AL (*β* = 0.14, SE = 0.04, 95% CI [0.073, 0.222]) (see Table 3). These results showed that TFL has a greater direct and indirect effect on work engagement than AL.

**Table 3:** Mediation models: Total, direct, and indirect effects and relationships among model variables.

Effect	*β*	SE	*p*	95% CI [lower, upper]		
Total	0.35 (0.46)	0.08 (0.07)	< 0.001 (< 0.001)	[0.226, 0.539] ([0.311, 0.594])		

Effect	*β*	Bootstrap SE		Bootstrap 95% CI [lower, upper]		
Indirect	0.14 (0.18)	0.04 (0.04)		[0.073, 0.222] ([0.111, 0.265])		

Model AL	Consequent					
Antecedent	Vigor at work			Work engagement		
	β	SE	*p*	β	SE	*p*
AL	0.29	0.06	< 0.001	0.21	0.07	0.002
Vigor at work	–	–	–	0.49	0.08	< 0.001
CV^1^	0.04	0.12	0.566	0.02	0.13	0.678
CV^2^	0.06	0.01	0.495	0.05	0.01	0.446
CV^3^	0.07	0.13	0.322	−0.15	0.13	0.011
CV^4^	0.09	0.01	0.259	0.01	0.01	0.845
Constant	*4.18	0.46	< 0.001	*0.74	0.58	0.209
	${R}^{2}=$ 0.09 F (5, 209) = 3.508, *p* = 0.005			${R}^{2}=$ 0.37 F (6, 208) = 19.972, *p* < 0.001		

Model TFL	Consequent					
Antecedent	Vigor at work			Work engagement		
	β	SE	*p*	β	SE	*p*
TFL	0.42	0.06	< 0.001	0.27	0.07	< 0.001
Vigor at work	–	–	–	0.44	0.08	< 0.001
CV^1^	0.04	0.11	0.535	0.03	0.12	0.609
CV^2^	0.08	0.01	0.334	0.07	0.01	0.314
CV^3^	0.07	0.13	0.346	− 0.15	0.12	0.007
CV^4^	0.13	0.01	0.122	0.04	0.01	0.605
Constant	*3.59	0.47	< .001	*0.61	0.55	0.271
	${R}^{2}=$ 0.18 F (5, 209) = 6.749, *p* < 0.001			${R}^{2}=$ 0.39 F (6, 208) = 23.956, *p* < 0.001		

In the effect row, the values outside the parentheses are those from the AL model, whereas values in parenthesis pertain to the TFL model. Bootstrap = 5.000. β = Standardized coefficient; CI = Confidence interval; CV = Control variable; CV^1^ = Gender; CV^2^ = Age; CV^3^ = Sector; CV^4^ = Tenure in the organization; SE = Standard error; AL = Authentic leadership; TFL = Transformational leadership.

* Unstandardized coefficient.

Moreover, AL and TFL were positively and significantly related to vigor at work. However, the impact of TFL on vigor at work was greater (*β* = 0.42, SE = 0.06, *p* < 0.001) than that of AL (β = 0.29, SE = 0.06, *p* < 0.001). Therefore, vigor at work was positively related to work engagement in both models (AL: *β* = 0.49, SE = 0.08, *p* < 0.001; TFL: *β* = 0.44, SE = 0.08, *p* < 0.001) (see Fig. 1).

The AL model explained 9% of the variation in vigor and 37% of the variation in the engagement at work, whereas these results were better for TFL, which explained 18% and 39% of the variation, respectively. Thus, TFL has a better overall result on vigor and engagement at work (Hypothesis 3).

## Discussion

Our study empirically extends our understanding of the relations that two positive leadership styles, AL and TFL, which include the ethical aspect of being a leader and that are considered to be job resources, have on a personal resource, such as vigor at work, and a motivational result, such as work engagement, in employees. These relations have been partially addressed in previous studies by analysing the impact of TFL or AL on engagement but not on vigor at work. Furthermore, there is a dearth of studies including employees´ perceptions of both styles of leadership in the same study.

Our results support our hypotheses, as vigor and engagement at work have shown to be related (Hypothesis 1). Moreover, our study shows that vigor mediates the effect of both leadership styles on engagement (Hypothesis 2). Furthermore, TFL relates to a higher engagement and has a bigger effect on the mediation of vigor concerning both leadership styles than does AL on work engagement (Hypothesis 3). Thus, the contribution of this research is twofold. First, vigor, as an affective dimension, mediates the effect of positive leadership on engagement, and second, in agreement with the meta-analysis conducted by Hoch et al.^39^, the contribution of AL beyond that of TFL in the context of energy constructs is also limited. Other studies have previously indicated that positive leadership may share a common ground regarding their effect on employee work engagement^51,88^. Our study indicates that the TFL considered as a positive leadership contributes to employees´ energy beyond AL which is also a genuine positive leadership. Thus, further analyses comparing different positive leadership styles could help to decide which could be of higher relevance for instilling energy in the organizations.

These results also have theoretical and practical implications. First, further comparisons among different positive leadership styles in the same study could shed light on the differential contribution of the components of leadership styles to promote one integrative proposal. As for the leadership styles analysed in this study, both AL and TFL are positively perceived by employees as creating a climate of energy that acts as a resource (both job and personal resources) that could explain the further relations of engagement to performance and job satisfaction^89^ and those of engagement and vigor at work to employees’ health^41^. This could help practitioners to focus interventions on improving leaders´ ways of energizing employees and further enhancing organizational and personal results. Our findings imply that transformational leaders to a higher extent than authentic leaders are the key ingredients for energizing employees, thus fuelling emotions/affect and motivation.

Another contribution of this study is that it allows to differentiate between vigor at work and engagement. Due to the influence and commonly used notion of engagement^90^, the term vigor has been absorbed by this approximation, although the proposal of Shirom implies a different concept, as defended in this research. Engagement is a persistent and pervasive affective—a cognitive state which includes vigor as high levels of energy and mental resilience while working, the willingness to invest effort in one’s work, and persistence even in the face of difficulties. Vigor at work clearly differs from the vigor dimension of work engagement, as people can experience vigor at work regardless of their psychological recovery capability to cope with adverse events (resilience and motivation^41^).

This study should be considered a starting point for analysis, as it is not devoid of limitations. In conducting EFA and CFA we show that both constructs are different, but there is one item overlapping in both scales. Thus, confusion about the energy displayed at work as a mainly psychological or physical aspect remains. In discarding the item, we focus on the basic concept avoiding artefacts. As demonstrated by Kulikowsky^90^ there are ambiguous results from studies focusing on the UWES factorial validity challenging the whole concept of work engagement as a three-factor structure of dedication, vigor and absorption. Instead, this author recommends to consider engagement as one dimension, as in this study, to better capture this motivational concept. Other authors suggest to include physical engagement, and not vigor, as a dimension to differentiate from UWES which implies a “broader attitudinal construct intimately related to employees’ well-being at work”^91^^(p.891)^. As a result, we further recommend to differentiate these two concepts, or even to deepen on an integrating concept that includes the cognitive-motivational and physical-affective aspects.

Another limitation refers to the sample size which prevents us from making further analyses that need a larger number of participants to make final conclusions (i.e. including all the variables in just one mediational or path analysis). Furthermore, this study was a cross-sectional and prospective study to analyse the possible relations among constructs. However, we could analyse the impact of different leadership styles on an energy resource and a motivational variable related to the well-being of employees^42^. For future research, to reduce common method variance, it would be interesting to collect measures from different sources or even to analyse these relationships by using a daily or longitudinal design. In this vein, measurement of the same sample at different times could help to determine whether these relations are maintained over time. Also note that we proposed a model in which the aim was to analyse the possible effect of two leadership styles (TFL and AL) on employee´s energy (vigor at work and engagement), but the design could also be reversed. As proposed in the JD-R theory, positive gain spirals imply that engagement promotes positive perceptions of resources (i.e. leadership styles) via job-crafting. In this case, energetic people could evaluate their leaders´ style more positively due to this positive state.

Finally, the sampling method means that most of the sample comes from private organizations (67%). Research about leadership and engagement has been usually conducted in private organizations whereas the focus on public or mixed organizations, although growing, remains limited^92^. For future studies, it will be of interest to deepen on other possible factors that can play the role of a moderator as, for example, the organizational context (private/public/mixed) which may influence the organizational culture.

## Data Availability

The datasets used and/or analysed during the current study available from the corresponding author on reasonable request.
